# AI-based system for food and beverage selection towards precision nutrition in Indonesian restaurants

**DOI:** 10.3389/fnut.2025.1590523

**Published:** 2025-04-25

**Authors:** Kudang Boro Seminar, Evy Damayanthi, Karlisa Priandana, Harry Imantho, Bonang Waspadadi Ligar, Annisa Utami Seminar, Angga Dwi Krishnajaya, Muhamad Reza Aditya, Muhammad Ilham Hakim Suherman, Ismy Fana Fillah

**Affiliations:** ^1^Faculty of Agricultural Engineering and Technology, IPB University, Darmaga Campus, Bogor, West Java, Indonesia; ^2^Faculty of Human Ecology, IPB University, Dramaga Campus, Bogor, West Java, Indonesia; ^3^School of Data Science, Mathematics and Informatics, IPB University, Dramaga Campus, Bogor, West Java, Indonesia; ^4^Faculty of Industrial Engineering, Gunadarma University, Depok, West Java, Indonesia; ^5^Karimata Restaurant, Bogor, West Java, Indonesia

**Keywords:** genetic algorithm, food selection, intelligent systems, restaurant, personalized nutritional status

## Abstract

The complexity surrounding food selection is attributable to the variability in foods, restaurants, and diners. The diversity of foods, where each dish may have a unique recipe across different restaurants, needs to be accounted for in personalized nutrition. However, personalized food selection poses a combinatorial challenge in selecting the most suitable food at a specific restaurant. The key question is how a diner visiting a particular restaurant can be assisted in selecting optimal foods and beverages based on factors such as sex, age, height, weight, and history of non-communicable diseases (NCDs). In this study, a genetic algorithm (GA) is used to develop a system that can address this issue in the context of Indonesian restaurants. In this system, a database with data on registered diners and foods is maintained. Foods comprise staple foods, side dishes, vegetables, and beverages, each containing its energy and nutrient content for a given restaurant. The nutritional adequacy of a single meal is determined by comparing the energy and nutrient content of the menu with the diner's nutritional needs. The novelty of the proposed system lies in combining scientific nutritional data with individual diner profiles for the selection of the best meal for a diner. This system differs from the existing food recommender applications in Indonesia, which typically do not consider specific diners, personalized nutrition, and NCD history. The proposed system is the first developed application prototype for Indonesian restaurants to overcome the inefficiency of the existing applications. In this study, the structure and chromosome content of the food, its corresponding energy and nutrient contents, and GA operators such as crossover, mutation, and tournament selection for determining the best meal using the defined fitness functions are discussed. The proposed system has been tested at Karimata Restaurant and proved to be highly suitable for the ultimate goal of meal selection for individual diners with different needs, and it can be replicated at other restaurants. Furthermore, user-centered evaluation has revealed that the system (a) increases nutritional understanding and health awareness; (b) is easy to use with comprehensive functions; and (c) promotes user satisfaction with personalized recommendations.

## Introduction

Food scientists and researchers have studied food choices in different contexts and perspectives (1–3). Studies have clearly shown that understanding what and why people eat and drink, and what they do about it, requires an understanding of various factors that may influence people's food and beverage choices and needs. Understanding individual-based motives that determine food choices is necessary to design the best interventions and support healthy food systems that cater to specific diner characteristics and preferences (2). Recently, diversity in the food and beverage industry has been increasing. The goal of food system transformation is to develop a future where everyone has access to healthy food prepared in sustainable and resilient ways that do not affect the environment and provide fair and equitable livelihoods. People need to gain a higher level of knowledge and awareness to choose healthy foods that suit their needs (4). The Food Is Medicine Institute envisions a world where consumption of nutritious food is recognized as a fundamental component of health and healthcare and where all people and communities have the knowledge, resources, and support to achieve optimal health and health equity through food (5).

Of late, the global food system and environment have changed dramatically, with changes in over- and undernutrition. In Indonesia, the diversity of foods served in restaurants has increased. Appropriate, healthy, and diner-specific food choices are becoming an increasing priority for human survival. According to precision nutrition, individuals may respond differently to certain foods and nutrients, so the best diet for one individual may be highly different from that for another. The science of food choice generates knowledge about the drivers of the food decision-making and behavior in the immediate food and social environment (6). Dietary recommendations using precision nutrition insights are based on principles more than just genetics. This approach incorporates elements such as age, sex, ethnicity, health history, lifestyle, eating habits, attitudes toward food, physical activity, microbiome, and metabolism. Therefore, precision nutrition provides dietary recommendations that consider these specific factors and offers a targeted method for preventing or managing chronic diseases (7, 8). Research on precision nutrition uses personalized information to provide tailor-made nutritional and dietary advice. Furthermore, machine learning, a branch of artificial intelligence, shows promise in building predictive models for precision nutrition (6).

The diversity in foods with various compositions of ingredients and concoctions in various restaurants necessitates that diners with specific conditions (sex, age, weight, height, and health history of NCDs) need guidance to select the best food. Moreover, Indonesia is required to increase the extent of food diversity and fortification to prevent a nutritional crisis (9). Food diversification, part of the government's efforts to minimize socioeconomic disparities with sustainable food development programs, can help utilize the potential of food resources completely (9). Another challenge to the healthcare industry is that eating habits have become worse around the world (10). Research has discussed the increase in advertising for junk foods and how some countries have started to protect children from consuming junk foods. Among the 11 million deaths attributed to poor diet every year worldwide, the leading cause is cardiovascular disease, which is often caused or made worse by obesity (10). Previous studies have developed some models of food recommender applications for Indonesian restaurants (11–14), but none of these applications has taken into account personalized nutrition, calculated and informed nutrition contents on foods, and NCD problems. Awareness of consuming food with balanced nutrition and according to energy needs should be fostered in society. Furthermore, this awareness forms a positive attitude toward nutrients and supports a healthy lifestyle (15).

This study aims to develop a smart application prototype that helps diners select Indonesian food and beverages provided by a restaurant. The aim of this study is to develop an intelligent model that helps diners select foods and drinks in specific restaurants based on precision nutrition, considering the increasing diversity of foods and restaurants in Indonesia. Previous studies have shown that research on food selection and meal planning recommendation algorithms has most extensively focused on the properties of food, specifically its nutrient composition (16, 17). The application developed in this study is a decision support system (DSS) that prioritizes food choices provided by restaurants and helps diners select the food choices that best suit their tastes and specific conditions.

## Materials and methods

### Materials: data and foods

The data used in this study were from Indonesian Food Composition Table (TPKI) released by the Ministry of Health, Republic of Indonesia, which contains food coding systematics, food nutrient/component systematics, food nutritional values, and edible weight (18). The supplemental data were from the NutriSurvey website, which contains several programs for nutrition calculations and surveys (19). This site aims to make nutrition calculations and surveys as user-friendly as possible and to keep the programs small and easy to install. One of the main NutriSurvey programs is the English version of a commercial German software (EBISpro) and is free for non-commercial use. All food data were obtained from our research partner Karimata Restaurant, namely types of meals and beverages, weight of portion, edible portion, ingredients of meals and beverages, serving size in one portion of meal, processing techniques, and price. The nutritional content of foods and their energy and nutrient adequacy were analyzed by a dietitian and a nutritionist.

In the data analysis and preprocessing phase, the composition of each menu was investigated and labeled based on laboratory analysis and the TKPI. All factors related to NCDs were included based on the nutritional guidelines for dietitians (20–23). The energy and nutrient content of the food consumed was calculated using the database from the TKPI, Nutrisurvey, and nutritional information on foods and beverages. The energy and macronutrient content of foods was calculated as follows:


$$\begin{array}{l} NC_{ij}=\;\frac{W_{j}}{100}\;*\;N_{ij}\;*\;\frac{PFI_{j}}{100}, \end{array}$$

where *NC*_*ij*_= nutrient content *i* in food ingredient *j*, *W*_*j*_ = weight of food *j* consumed (grams), *N*_*ij*_ = nutrient content *i* in 100 g edible portion of food ingredient *j*, and *PFI*_*j*_ = percent of food ingredient *j* that can be eaten.

The calculated energy and nutrient content was then used to calculate the adequacy level, which is represented by the nutrient adequacy ratio (NAR). The NAR is a measure of how well an individual's intake of a specific nutrient meets the recommended daily allowance (RDA). The RDA is the average daily intake level of a nutrient that is sufficient to meet the nutrient requirements of nearly all (97–98%) healthy individuals of a specific age, sex, and lifestyle. The NAR is expressed in percentage and helps assess dietary sufficiency for each nutrient, which is calculated as follows (24):


$$\begin{array}{l} NAR=\left(\frac{Nutrient\;consumption}{RDA\;of\;nutrient}\right)\times \;100\%. \end{array}$$

According to Widyakarya Nasional Pangan dan Gizi (WPNG) (25), the NAR is divided into five categories, namely severe deficit (< 70% RDA), moderate deficit (70–79% RDA), light deficit (80–89% RDA), sufficient (90–119% RDA), and more than sufficient (≥120% RDA).

### GA representation

The outcome of this research is a DSS application model equipped with a genetic algorithm (GA) computational framework for selecting meals based on basic dish, side dishes, vegetables, and beverages available at Karimata Restaurant. The menu options included 1 basic dish (rice), 33 side dishes, 18 vegetable dishes, and 24 beverages. The GA structure used in this system was enhanced from its previous design, which included only the elicitation operation (23). The current version, based on recent research, incorporated crossover, mutation, and tournament selection operations (26).

The GA framework was also used to address the potential conflicts between a diner's nutritional requirements and the limitations of available menu items by implementing a penalty-based fitness function evaluation method that accounts for deviations from nutritional targets. Instead of eliminating menu items that do not perfectly match the dietary requirements, the model assesses the degree of discrepancy for key nutritional components, such as caloric content, protein, sodium, and fat levels. Each deviation is assigned a penalty score, quantifying its discrepancy from the ideal. These penalties are then aggregated to compute the total penalty value for each meal configuration. This total penalty value is normalized to produce a fitness score ranging from 0 to 1. A lower cumulative penalty indicates greater nutritional alignment and results in a higher fitness score, thereby increasing the probability of that meal being selected. In contrast, larger deviations yield higher penalty scores, which reduces the likelihood of recommendation. This framework addresses practical limitations by allowing suboptimal yet feasible options. By prioritizing meal combinations that minimize overall nutritional disparity, this model facilitates personalized meal selection that strikes a balance between theoretical optimality and real-world availability.

From a GA perspective, food and its nutritional content form a chromosome that comprises 13 genes (Table 1), in order as follows: basic dish (rice), side dishes, vegetables, beverages, calories, proteins, carbohydrates, sodium, cholesterol, saturated fatty acids (SFAs), monosaturated fatty acids (MUFAs), and polyunsaturated fatty acids (PUFAs). The GA framework used in this system considers diner-specific factors such as sex, age, weight, height, and NCD history. The developed GA model matches restaurant menu items from four food groups: (1) basic dish, (2) side dishes, (3) vegetables, and (4) beverages, with respect to the energy and nutrient requirements of each diner.

**Table 1:** Chromosome structure representing foods (gene 1–4) and energy and nutrient content of the food (gene 5–13).

**Gene 1**	**Gene 2**	**Gene 3**	**Gene 4**	**Gene 5**	**Gene 6**	**Gene 7**	**Gene 8**	**Gene 9**	**Gene 10**	**Gene 11**	**Gene 12**	**Gene 13**
Basic dish	Side dish	Vegetable	Beverage	Energy (Cal)	Protein (gram)	Fat (gram)	Carbohydrate (gram)	Sodium (mg)	Cholesterol (mg)	MUFA (gram)	PUFA (gram)	SFA (gram)

The operations crossover, mutation, and tournament selection are applied to gene 1 (basic dish), gene 2 (side dishes), gene 3 (vegetables), and gene 4 (beverages), which are presented in Table 1. The final composition of gene 1 to gene 4 is based on the final minimum difference between the nutritional content (gene 5 to gene 8) of food and the diner's one-meal nutrient requirement. As shown in Table 1, the nutritional content of gene 9 to gene 13 is the maximum content level permitted for diners with a history of diabetes, hypertension, and coronary heart disease.

**Figure 1:**
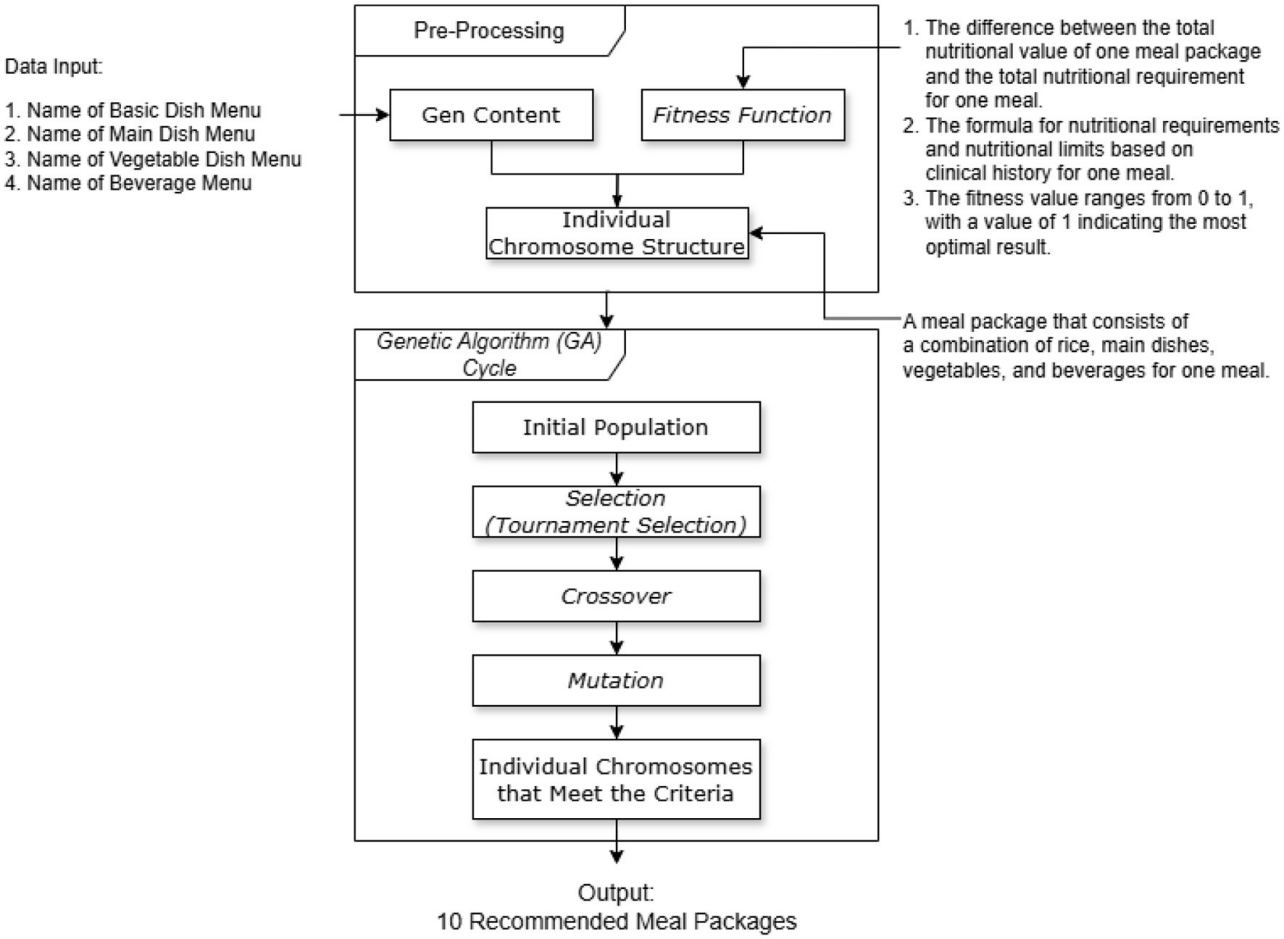
GA model flow for food menu package recommendations.

These operations enable the formation of the best chromosome that represents an optimal combination of menu items from the predefined categories gene 1–4. The “best chromosome” signifies the selected menu combination that contains the levels of macro- and micronutrients that are most closely aligned with 30% of a diner's daily nutritional needs (for a single meal). This is based on the assumption that the daily nutritional intake of a diner is distributed as follows: 20% for breakfast, 30% for lunch, 20% for snacks, and 30% for dinner. In addition, parameters such as sex, age, weight, height, and health history (healthy, or having one of the following conditions: diabetes, hypertension, or coronary heart disease) are considered for diners (27). The GA model flow for food menu package recommendations is shown in Figure 1.

### Preprocessing

The process begins with defining the content representation of the “genes” within a chromosome as data input. These genes typically represent variables or parameters for the optimization problem. The chromosome structure consists of gene 1 (basic dish), gene 2 (side dishes), gene 3 (vegetables), and gene 4 (beverage). The details of each gene are as follows:

- Gene 1: Represents basic foods such as rice. This gene will always be randomly selected from items at index 0 or 1 in the dataset: 0 represents with rice, and 1 represents without rice.
- Gene 2: Represents side dishes, which contain protein-based foods. This gene is randomly selected from a broader range of items (index 2–34) from the dataset.
- Gene 3: Corresponds to vegetables and is randomly selected from items indexed between 35 and 52 in the dataset.
- Gene 4: Represents beverages and is randomly selected from items indexed between 53 and 78 in the dataset.

**Figure 2:**
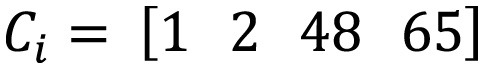
Individual chromosome structure. *C*_*i*_ corresponds to the *i*-th individual chromosome. Values 1, 2, 48, and 65 in each gene represent in order rice, grilled catfish in bamboo, tofu soup with vegetables, and watermelon juice.

These genes and ranges imply that the dataset is organized categorically and that each gene corresponds to items within a specific category. The example of an individual chromosome is shown in Figure 2.

The fitness function is a critical element that evaluates how “fit” (or good) an individual chromosome is in solving the problem. It assigns a numerical value to each chromosome, with higher values representing better solutions. In this study, the fitness function is calculated by comparing an individual's nutritional profile with predefined nutritional needs. The nutrients considered in this study are shown in Table 2.

**Table 2:** Nutrients of the foods.

Energy (cal)	Protein (g)	Fat (g)	Carbohydrate (g)	Fiber (g)	Sodium (mg)	Cholesterol (mg)	MUFA (g)	PUFA (g)	SFA (g)

To calculate the fitness function, the penalties of each nutrient corresponding to each dish need to be identified. This penalty-based system quantifies deviations from optimal nutritional targets and adjusts for clinical conditions. The higher the penalty for a dish, the lower the probability of being recommended. In short, a dish with a high penalty value does not match the predefined nutritional needs of a specific diner. The formula used to calculate the penalties is as follows:


$$\begin{array}{r} penalty_{i}=\left[actual\;nutrition\left[i\right]-nutrional\;needs\left[i\right]\right],\;\forall i\\ \in \left\{0,1,2,\ldots ,9\right\} \end{array}$$

where

**Figure 3:**
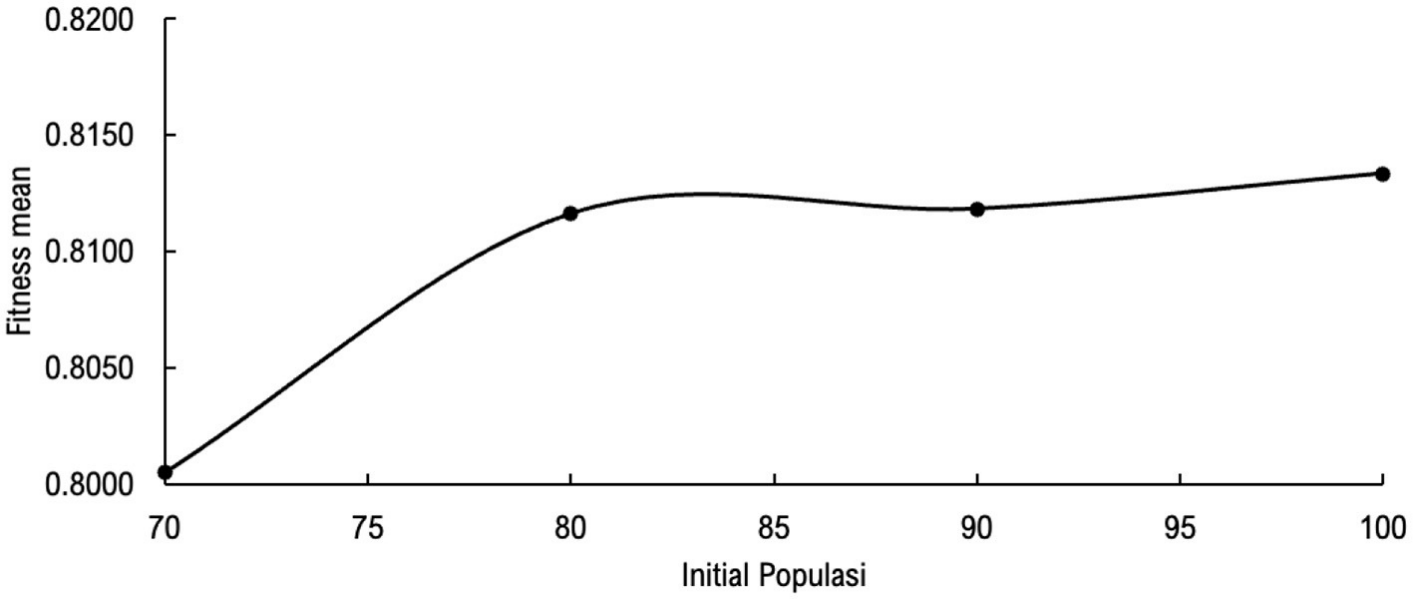
Effect of the combination of the initial population, mutation probability, and crossover probability on the best fitness mean value.

- *actual nutrition*[*i*] is the actual value of the *i*-th nutrient,
- *nutrional needs*[*i*] is the target value of the *i*-th nutrient,
- *penalty*_*i*_ is the penalty of the *i*-th nutrient, and
- ∀*i* ∈ {0, 1, 2, …, 8} refers to the nutrients displayed in Figure 3.

This formula can be used for people with no medical conditions because for people with medical conditions, the formula should be specific to each medical condition (23).

Next, the total penalty or the fitness value is calculated. It is the sum of individual penalties for all nutrients. This provides an aggregate measure of how well the individual's nutritional profile meets the targets. The formula to calculate the total penalty is as follows:


$$\begin{array}{l} total\;penalties\;\left(Fitness\;Value\right)=\overset{9}{\underset{i=0}{\sum }}penalty_{i} \end{array}$$

where

- *i* represents the index of each nutrient (e.g., 1 for energy, 2 for protein, and so on),
- the summation runs from *i* = 0 *to i* = 9, covering all nine nutrients (energy, proteins, fats, carbohydrates, sodium, cholesterol, MUFAs, PUFAs, and SFAs), and
- *penalty*_*i*_ is the penalty of the *i*-th nutrient.

The next step is to normalize the fitness value obtained from the total penalty formula to a score between 0 and 1. Individuals with no penalties achieve a fitness score of 1 (highest fitness), whereas individuals with maximum penalties achieve a score near 0 (lowest fitness). The formula used is as follows:


$$\begin{array}{l} normalized\;fitness=1-\frac{\left(total\;penalties\;-\;\min\;penalty\right)}{\left(\max\;penalties\;-\;\min\;penalty\right)} \end{array}$$

where

- max *penalties* is the maximum value of penalties (1,000), and
- min *penalty* is the minimum value of penalties (0).

The maximum penalty value was chosen arbitrarily (not absolutely) as a number large enough to accommodate various levels of penalty. It is used as the upper limit of total penalty that can be given to an individual with very bad nutritional profile. Because the fitness range is 0–1, if the penalty is 1,000, the fitness is 0 (very good nutritional profile); conversely, if the penalty is 0, the fitness is 1 (very bad nutritional profile). If the penalty is smaller than the maximum penalty, the fitness value will approach 1; conversely, if the penalty is too large, then the fitness value will approach 0. This makes distinguishing between individuals with good and bad nutritional profiles difficult.

The normalized fitness formula returns the fitness score, which quantifies how closely the individual's nutritional profile aligns with the predefined nutritional requirements, taking into account condition-specific penalties. A fitness score closer to 1 indicates better alignment and suitability.

### Genetic algorithm cycle

#### Initial population

The GA cycle begins with a randomly generated population of chromosomes, which serves as the foundation for exploring the solution space. This initial randomness ensures that the algorithm can investigate a broad range of possibilities without any influence by prior biases. Each individual chromosome in the population represents a potential solution to the problem at hand, forming the initial search space.

As the population evolves, its progression is guided by fitness evaluations and genetic operators, with the diversity and quality of the initial population playing a pivotal role in the algorithm's performance. A diverse population provides a broader genetic pool, which enhances the likelihood of discovering optimal solutions, whereas insufficient diversity comes with the risk of premature convergence to suboptimal results (28). The steps for generating populations are as follows:

- Initialization: Creating the initial population of individual chromosomes, which serves as the starting point for the evolutionary process.
- Intermediate Populations: Generating new populations during iterative stages of the algorithm, such as after applying selection, crossover, and mutation operations.

The best initial population parameters, along with mutation probability (mp) and crossover probability (cp), were determined using a random search method. The best fitness mean value was used as a reference in selecting the three model parameters. Initial population, mp, and cp were determined randomly: [70, 80, 90, 100], [0.0001, 0.0002, 0.0005, 0.067], and [0.8, 0.85, 0.9, 0.95], respectively. Figure 3 shows the effect of initial population on the best fitness mean value, in combination with mp [0.0001, 0.0002, 0.0005] and cp [0.8, 0.85, 0.9]: the larger the selected initial population value, the more stable the fitness mean value, but the longer the GA model will take to produce the best chromosomes.

#### Selection (tournament selection)

Once the initial population is generated, chromosomes that will participate in reproduction are identified in the selection process. Tournament selection is one of the most widely used selection methods. It involves selecting a small subset of chromosomes (a “tournament”) from the population and comparing their fitness values. The chromosome with the highest fitness value within this subset is chosen as a parent for the next generation. In addition, the selection process ensures that better-performing chromosomes have a higher likelihood of contributing their genetic material to the offspring. This mechanism promotes the principle of “survival of the fittest,” where stronger candidates are more likely to reproduce, whereas weaker candidates are gradually eliminated.

**Figure 4:**
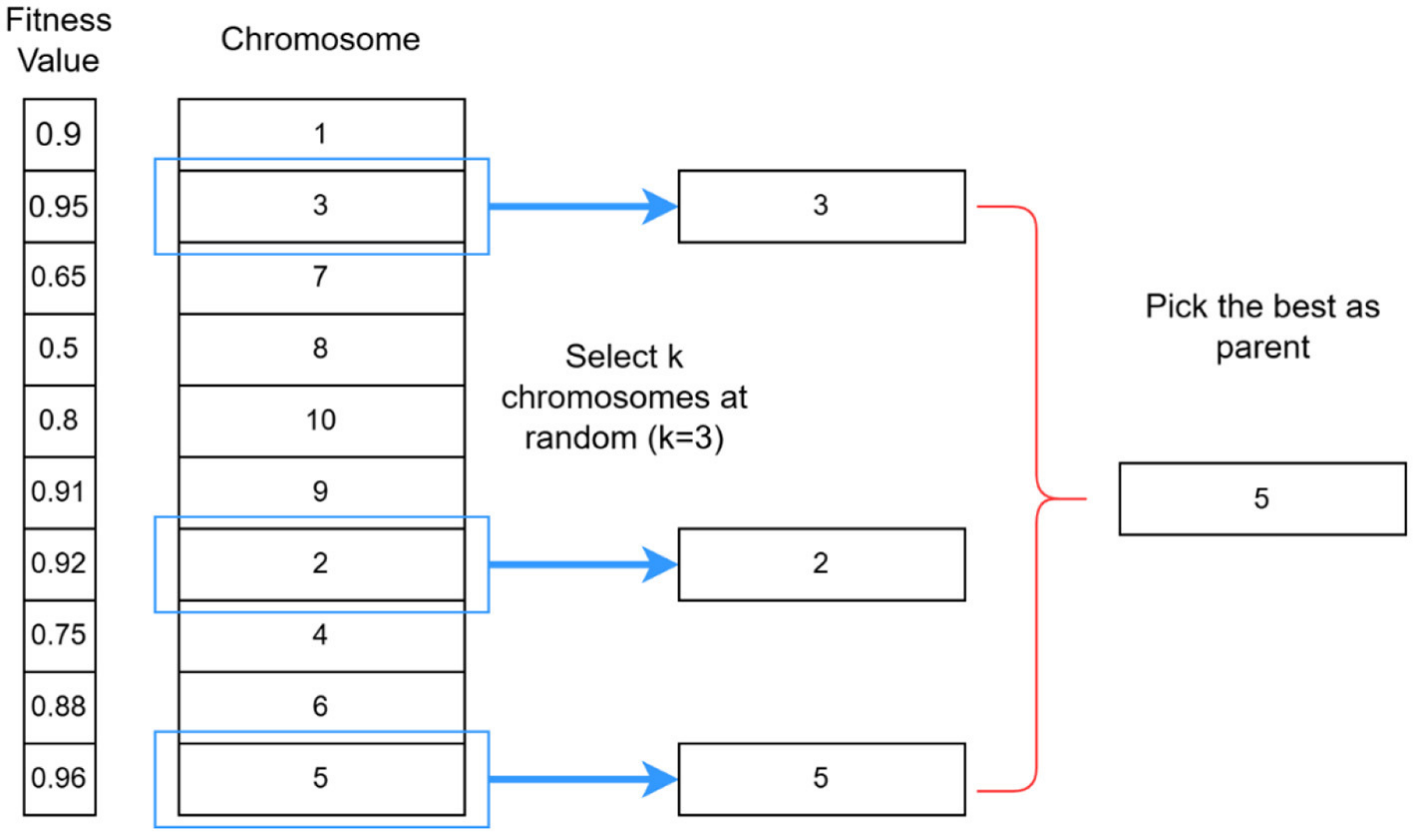
Illustration of the tournament selection process.

As shown in Figure 4, in the tournament selection process, *k* individual chromosomes are randomly chosen from the population to compete in a tournament, and the individual chromosome with the highest fitness value is selected to advance to the next generation. This tournament process is repeated until the new population is completely filled (29). The tournament begins by randomly selecting a group of individual chromosomes from the population. For example, in the first tournament, individual chromosomes with fitness values of 0.9, 0.95, and others are chosen. The individual chromosome with the highest fitness value within the subset, in this case 3 (fitness value 0.95), is declared the winner and it moves on to the next stage. This process is repeated for subsequent tournaments. In the second tournament, individual chromosome 2 with a fitness value of 0.92 is declared the winner, and in the third tournament, individual 5 with a fitness value of 0.96. These winners form the pool of candidates for the next generation. The tournaments continue until the new population is completely filled with individual chromosomes selected based on their fitness values.

#### Crossover (recombination)

Crossover is a fundamental operation in GAs that emulates biological reproduction by merging the genetic material of two parent chromosomes to create offspring. This process facilitates the exploration of new regions in the solution space and enables the generation of novel gene combinations. By introducing diversity into the population, crossover helps prevent the algorithm from stagnating in local optima and enhances its ability to find optimal solutions (30). A random decision, guided by a cp, determines whether a gene is exchanged at a specific position (31). The types of the crossover method include uniform crossover, single-point crossover, and multipoint crossover (32).

#### Mutation

Mutation introduces randomness into a GA population by altering one or more genes in a chromosome. By introducing slight variations in the genetic makeup of individual chromosomes, mutation enables the exploration of new areas in solution space (28). Mutation typically involves randomly selecting a gene within a chromosome and modifying its value, with the nature of the modification depending on the encoding scheme. Binary encoding may involve flipping a gene from 0 to 1 or vice versa, while real-number encoding might adjust the gene's value incrementally. These modifications are usually applied with a low probability to ensure stability while encouraging the exploration of new possibilities (33).

#### Output (individual chromosomes that meet the criteria)

The process of identifying the best-performing chromosomes in a GA involves multiple considerations to ensure that the solutions generated meet the predefined optimization criteria. These chromosomes represent either optimal or near-optimal solutions to the problem being addressed, as determined by their high fitness values. The culmination of the iterative process in a GA highlights these top-performing individuals, which encompass the best combinations of genes to solve the problem effectively.

The key elements in output selection are as follows:

1. Termination Criteria:
2. The algorithm's iterations may terminate based on several conditions:

• Maximum Generations Reached: The algorithm stops after completing a predefined number of iterations.
• Target Fitness Level Achieved: When the fitness value of the best chromosome surpasses a target threshold, the algorithm halts as it has found a satisfactory solution.
• Stagnation: If there is no significant improvement in fitness values over a set number of generations, the algorithm assumes convergence and terminates early.

2. Top Individuals:
The final output includes the best-performing chromosomes, typically ranked by fitness values. These chromosomes often represent unique combinations. Ensuring uniqueness is critical in domains such as menu optimization, where redundant solutions may lack practicality.

3. Statistical Analysis:
The algorithm also computes aggregate metrics such as mean fitness values of the top individuals. This provides a broader perspective on how well the population performed collectively and aids in understanding the algorithm's effectiveness.

4. Parameter Sensitivity:
The performance of the algorithm and, consequently, the quality of the output heavily depend on parameters such as Population size, cp, and mp. These parameters should be fine-tuned to ensure that the algorithm maintains a balance between exploration (finding diverse solutions) and exploitation (refining existing solutions).

• In this study, population sizes of 60, 70, and 90 were chosen to assess the trade-off between computational efficiency and genetic diversity in GA-based menu optimization. A smaller size (60) enables faster computation but risks premature convergence, whereas a larger size (90) enhances diversity and broadens the search scope, thus increasing the probability of identifying high-quality meal combinations that better satisfy personalized nutritional needs at the cost of longer runtimes. The intermediate size (70) serves as a balanced option, helping identify the optimal configuration for achieving high-quality personalized meal recommendations within realistic computational limits.
• This study explored four levels of crossover probability, 0.8, 0.85, 0.9, and 0.95, to comprehensively investigate the effect of varying recombination intensities on the performance of the GA in food and beverage selection. These values fall within a high-probability range that is widely recommended in the evolutionary computation literature for optimization tasks where both solution diversity and convergence are critical. Given the complexity of the menu recommendation problem, with multiple nutritional constraints personalized to each diner, the algorithm requires a thorough exploration of the solution space. High cps (80–90%) promote frequent recombination, enhancing diversity and helping the GA avoid premature convergence.
• To safeguard genetic diversity and limit the probability of early convergence on subpar solutions, mp was fine-tuned as well. Values such as 0.0002 and 0.067 were applied. A low mp of 0.0002 was used to maintain stable, well-optimized meal combinations by introducing only minimal changes, preserving nutritional integrity. In contrast, a higher rate of 0.067 resulted in broader exploration, helping the algorithm avoid early convergence by testing more diverse and potentially better menu configurations within complex dietary constraints. This method provided controlled randomness, allowing the algorithm to explore new possibilities while still retaining promising gene patterns.

All these parameters were explored together to identify the most effective combinations. The aim was to generate menu recommendations that scored high in both personalization and nutritional value, all within the bounds of what the restaurant could realistically offer. The results indicate that every time the GA model was executed, the best menu recommendations never yielded the same values for parameters, reflecting the algorithm's inherent stochastic nature. This randomness is a fundamental feature of evolutionary algorithms, which enabled the model to explore a broader solution space and reduced the likelihood of premature convergence to local optima. Such variability is particularly advantageous in complex multiobjective problems such as personalized meal selection, where diverse and adaptive solutions are essential.

## Results and discussion

In this study, an intelligent system has been successfully developed to assist diners in choosing foods with nutritional content that is appropriate to their nutritional requirements. This system uses biometric data (sex, age, weight, height) and history of NCDs, namely diabetes, hypertension, and coronary heart disease. While the Mifflin-St Jeor Equation (34) provides a foundational estimate for resting metabolic rate, total energy expenditure should incorporate standardized physical activity multipliers (e.g., 1.2 for sedentary lifestyles or men 1.65 and female 1.55) to account for daily movement without relying on time-intensive questionnaires. Profession-based activity tiers may offer a pragmatic proxy but require validation against population norms. For NCD management, macronutrient adjustments (e.g., controlled carbohydrates for diabetes, reduced sodium for hypertension) and culturally adapted meal plans should be prioritized to enhance adherence (35).

The technique of optimizing calorie and nutrient intake in the system provides food choices based on fulfilling consumers' calorie requirements and nutrient content in food. A crucial part of this GA-based DSS is the fitness function, which measures how well a food combination meets the nutritional needs of a consumer. The search for the best fitness value is carried out by applying selection, crossover, mutation, and tournament selection rules across generations, from gene 1 to gene 4 as shown in Figure 1. In this study, we were unable to use genetic data because in Indonesia genetic data are confidential and restricted to those who analyze them in the clinic. However, in the future, with the availability of wider public data collected by the Indonesian Ministry of Health, the results of this research can be adjusted.

### GA-based personalized food selection

The size of the initial population was a key factor in the GA-based model for providing food menu package recommendations. A larger population fostered genetic diversity and improved the chances of finding high-quality solutions though they came with increased computational costs. Conversely, smaller populations were computationally efficient but may limit diversity, hindering the algorithm's ability to thoroughly explore the solution space (36). Meanwhile, by striking a careful balance between population size and diversity, GAs can effectively navigate the solution space and evolve toward optimal outcomes with each generation (37). The population sizes used in the present study involved three scenarios, which were 60, 70, and 90. These different scenarios were used to compare and find the optimal solutions.

The crossover method used in this study was uniform crossover, where each gene in a chromosome was independently considered for crossover. Unlike single-point or multipoint crossover, in which entire segments of the chromosome at predefined locations were swapped, uniform crossover allows for localized gene swapping, fostering diversity and generating feasible solutions. This targeted approach balances between exploration and exploitation of the solution space, ensuring that the algorithm maintains diversity while refining promising solutions (32). Uniform crossover was widely applied beyond menu optimization and was also utilized in areas such as feature selection for classification, where it effectively combines relevant traits from parent chromosomes to improve solution quality (38). Its ability to integrate randomness while respecting problem-specific constraints makes uniform crossover a robust and essential component of modern GAs, which enhances its adaptability and performance across diverse applications.

**Figure 5:**
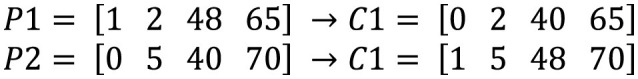
Illustration of uniform crossover (P = parents, C = child).

Three cp scenarios were used in this study, namely 0.8, 0.85, and 0.9, to determine the impact of different crossover intensities on the performance of the algorithm. For example, cp = 0.8 means that the cp will occur by 80% when two parent chromosomes are selected. Figure 5 illustrates the crossover process in gene 1 and gene 3, in two parents (P1 and P2).

**Figure 6:**
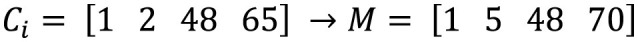
Illustration of the mutation process (M = mutated chromosome).

Mutation plays a crucial role in maintaining genetic diversity and preventing premature convergence, reintroducing variability. It enables the algorithm to explore parts of the solution space that may not be reachable through selection and crossover alone. In this study, the mutation process was applied independently to each gene, with the probability being determined by mp. A random value between 0 and 1 was generated to initiate the mutation process. If the value is less than mp (0.0002, 0.067), then a mutation will occur. As shown in Figure 6, gene 2 and gene 4 are genes that are mutated.

The fitness value of each new generation produced through the crossover process and mutation generation was calculated. The composition and nutritional content evaluated using the fitness function included macronutrients (gene 5 to gene 9) and micronutrients (gene 10 to gene 13).

Tournament selection was applied to choose individual chromosomes from the current population to act as parents for the next generation. A total of 10 chromosomes with the highest fitness value were selected from all individual chromosomes in the population. These ten chromosomes were the menu sets comprising staple foods, side dishes, vegetables, and beverages and met the consumer's nutritional needs. The developed GA model also returned optimal parameters, such as population size, cp, and mp. A total of 10 food and beverage recommendations generated by the GA-based model for a healthy male consumer (age 30 years, weight 70 kg, and height 170 cm) are shown in Table 3.

**Table 3:** Food and beverage recommendations generated by the GA-based model.

**No**	**Gen1**	**Gen2**	**Gen3**	**Gen4**	**Gen5**	**Gen6**	**Gen7**	**Gen8**	**Gen9**	**Gen10**	**Gen11**	**Gen12**	**Gen13**	**Fitness**
						**Energy (Cal)**	**Protein (g)**	**Fat (g)**	**Carbohydrate (g)**	**Sodium (mg)**	**Cholesterol (mg)**	**MUFA (g)**	**PUFA (g)**	**SFA (g)**
1	Nasi	Patin bakar kecap	Sup tahu + sayuran	Jus semangka	868	48.4	20.9	167.8	717.42	146.63	9.13	5.93	12.40	0.902
2	Nasi	Ayam goreng kremes	Sup sayuran	Jus mangga	846	22.8	34.9	158.3	756.66	109.66	10.87	5.63	19.70	0.849
3	Nasi	Patin bakar kecap	Brocoli bawang putih	Jus wortel	837	39.5	14.1	182.4	734.00	139.73	7.30	3.23	10.07	0.843
4	Nasi	Patin bakar dalam bambu	Terong sambal hijau	Jus stamina	990	52.0	38.7	157.2	717.52	82.50	1.29	2.40	9.07	0.828
5	Nasi	Dori crispy	Terong ikan asin	Es cendol	850	22.0	26.3	179.2	630.42	33.27	2.37	1.33	9.93	0.792
6	Nasi	Patin bakar kecap	Karedok	Jus munser	977	47.4	30.7	176.9	671.61	144.93	12.40	6.47	16.10	0.789
7	Nasi	Gurame goreng asam pedas	Cah kangkung terasi	Jus nanas	1,007	48.4	25.4	185.7	748.85	131.60	8.97	6.60	24.03	0.762
8	Tanpa Nasi	Gurame goreng asam pedas	Sup tahu + sayuran	Jus glucoless	815	45.2	34.6	82.0	790.62	137.77	10.43	9.03	26.10	0.749
9	Nasi	Sup gurame asam kemangi	Sup sayuran	Es lemon	794	41.2	9.2	179.7	652.15	137.77	6.27	3.53	6.90	0.746
10	Nasi	Udang saus asam pedas	Sup tahu + sayuran	Jus stamina	769	33.1	23.7	156.4	625.54	75.90	3.23	4.33	8.00	0.745

### Intelligent decision support system for selection prototype of foods and beverages in Indonesian restaurants

The intelligent DSS prototype for food and beverage selection in Indonesian restaurants was developed as a web-based application using a responsive design approach, which makes it easy to use on a variety of platforms (computers, tablets, and smartphones). The integration of object-oriented system development and microservice methodologies ensured that the application is resilient to the complexity and dynamics of rapid change in today's technological age. Representational State Transfer - Application Programming Interface (REST-API) technology was used to enable seamless communication between the GA-based model and the frontend and the backend.

**Figure 7:**
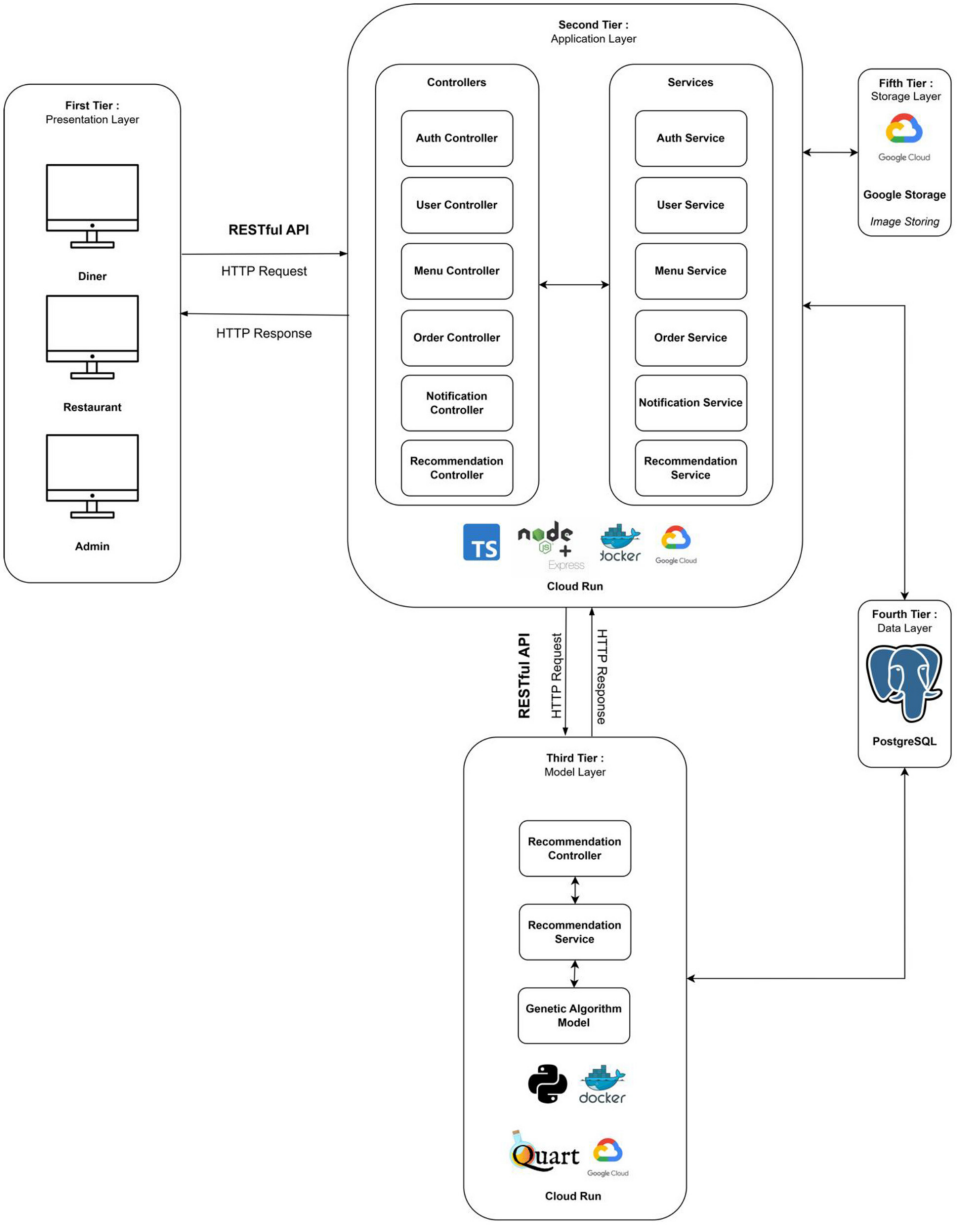
Architecture of the intelligent decision support system for the selection of food and beverages in Indonesian restaurants.

The developed system has a five-tier architecture, as shown in Figure 7. The first layer, the presentation layer, serves as an interface (frontend) to visualize data and information through a web-based application. The second layer, the application layer, processes the core business logic of the application system. It is also referred to as the backend application service, which was developed using TypeScript with the Node.js runtime environment and the Express.js framework. The third layer, the model layer, executes the computation of the GA model to generate personalized food and beverage recommendations. It is also known as the backend model and was implemented using Python with the Quart framework. The fourth layer, the data layer, stores and manages structured data using an ORDBMS, namely PostgreSQL. Finally, the fifth layer, the storage layer, is designed to store unstructured data, such as binary large objects, i.e., images, using Google Cloud Storage. Communication between these layers is facilitated through API requests using HTTP, which adheres to the RESTful API architecture in accessing resources from each layer.

**Figure 8:**
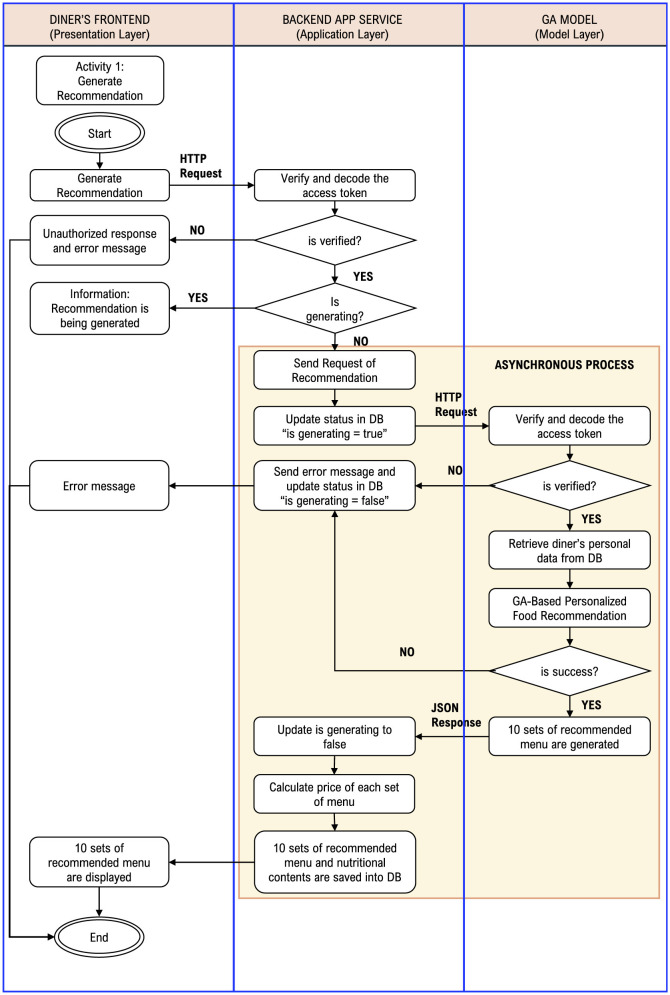
Activity diagram for generating 10 sets of recommendation menu.

The generation of recommendations begins with a request from the diner(s) via the frontend layer. The request is then validated and forwarded to the backend layer to run the GA model. The GA model calculates 10 personalized food and beverage recommendations based on the diner data stored in the database. The recommendations generated by the GA model are sent back to the backend application service, along with the total price and nutritional content description, and are finally displayed on the diner's frontend. This process is well illustrated in the activity diagram presented in Figure 8.

**Figure 9:**
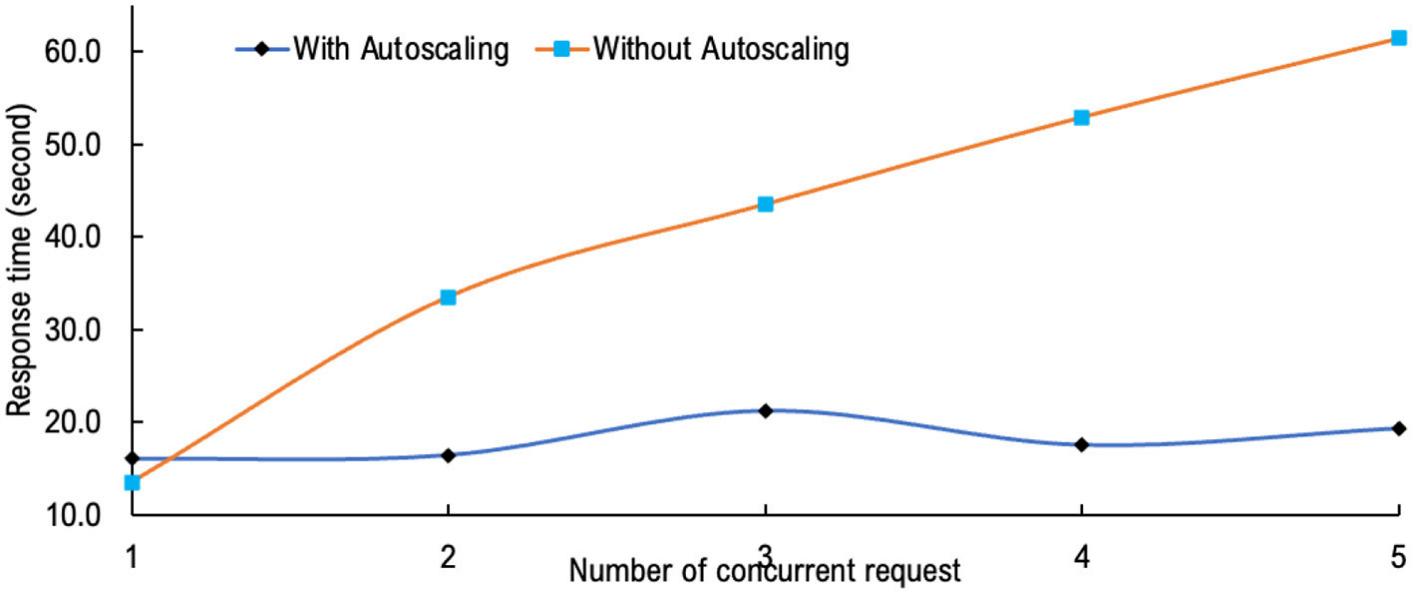
Response time testing of the application with and without autoscaling.

Response time testing is carried out on a production server using a Cloud Run serverless service with CPU specifications of eight cores up to 2.4 GHz and 16 GB RAM. The test results with and without autoscaling showed significant performance, as shown in Figure 9. For response time testing, K6 by Grafana with one to five concurrent requests/connections is used. The system is designed to optimize the autoscaling feature by implementing a parallel processing method on the model layer to respond to concurrent requests. The reliability of system performance in maintaining the response time for up to five concurrent requests is shown in Figure 9.

**Figure 10:**
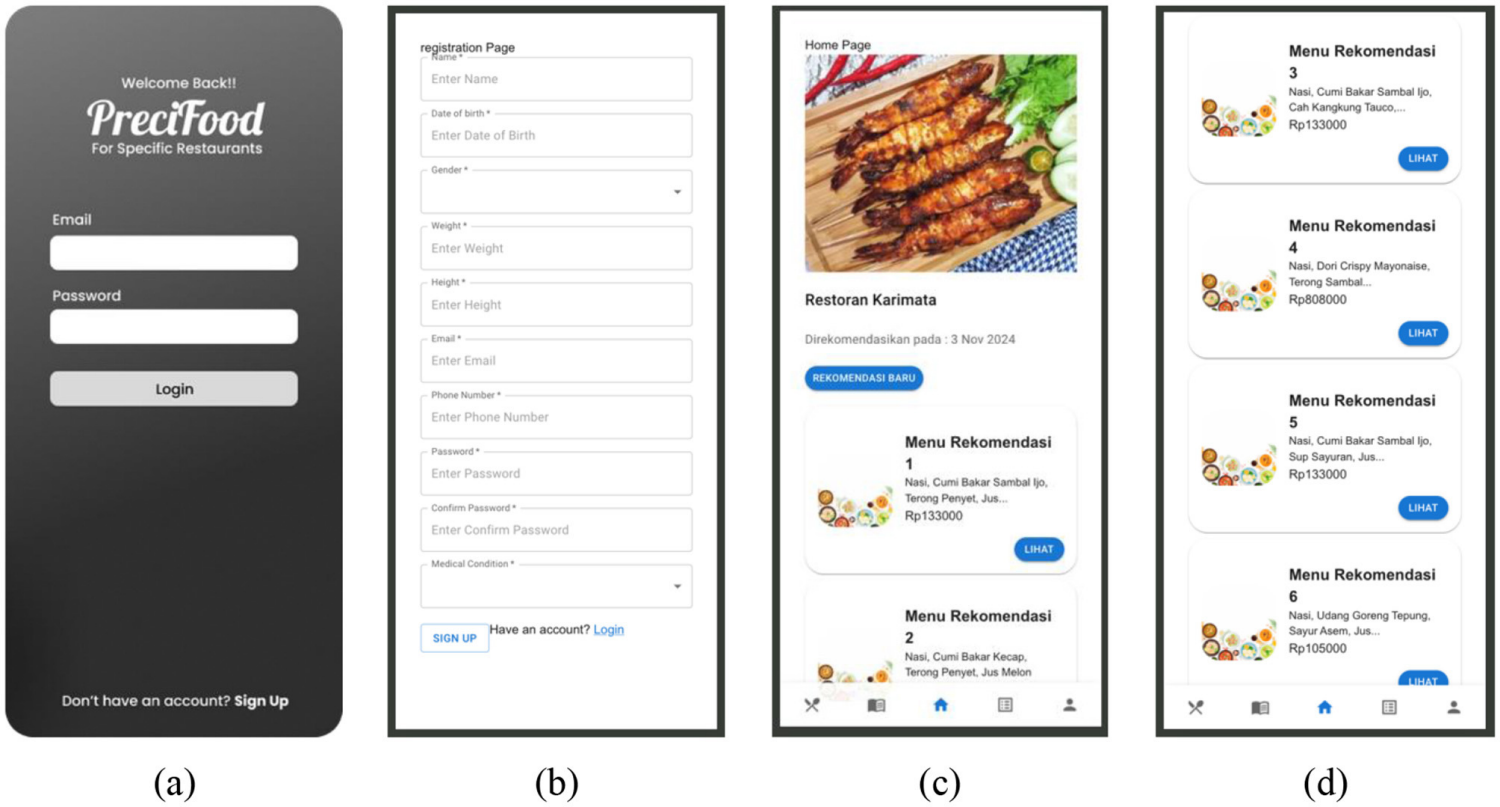
Application user interface. **(a)** the log-in page, **(b)** the registration page for new users, **(c)** the initial dashboard page after successful login, and **(d)** the menu recommendation page.

The system's interface is shown in Figure 10. The dashboard page (Figures 10c, d) is the initial page displayed to consumers after successfully logging in. On this page, consumers can see 10 recommended menu options generated by the GA model. This recommendation is computed based on the diner's personal data as soon as they successfully log in to the application. The output of the GA-based model consists of a combination of (1) basic dish (rice/without rice), (2) side dishes, (3) vegetables, and (4) beverages. The 10 recommendation menu sets are displayed on the frontend page (Figures 10c, d). Diners can explore the details of the menu recommendations by pressing the “VIEW” button and select a recommendation menu set by pressing the “ORDER” button. The order summary page contains the selected menu set and a summary of nutritional fulfillment. This information is extremely important as it indicates the actual amount of nutritional needs that are met, the nutritional elements, and the difference in deficiencies per nutritional element that is not met.

The system was tested using various menu sets at Karimata Restaurant. The results showed its effectiveness in generating personalized food menu recommendations for both healthy individuals and those with a history of NCDs. In addition, interviews were conducted with 15 diners at Karimata Restaurant who used the application prototype. The participants included 10 individuals aged 16–35 years and 5 individuals aged 40–60 years. These interviews yielded valuable insights for improving the application.

• Promotion of Nutritional Understanding and Health Awareness
- The application was perceived as a beneficial tool, particularly among health-conscious participants. These participants used the nutritional information provided, such as calories, protein content, and fat content, as a guide for managing their dietary intake. For participants who were less selective about their food choices, the application served as a source of new knowledge, reinforcing rather than altering their usual preferences. Participants with pre-existing diet plans found the application especially helpful as it automated the process of calculating daily calorie intake, which they previously performed manually. The application made decision-making regarding food selection significantly easier.

• Ease of Use and Feature Completeness
- The majority of participants agreed that the application was easy to use and offers a comprehensive set of features. The interface was especially well received by younger users who were familiar with digital technology. However, older users noted the need for initial guidance to navigate the application effectively. Suggestions included incorporating a tutorial or popup instructions to assist first-time users. Although the application was largely considered user-friendly, some technical issues were identified, such as slow loading times for menu recommendations and dependency on a stable Internet connection.

• Positive Impressions and User Satisfaction
- The overall user satisfaction was high. The availability of nutritional information was highlighted as a key benefit that supported informed food choices. Despite minor technical challenges, participants described their experience with the application as positive. Many expressed their willingness to recommend the application to friends and family.

• Perceived Innovation and Future Potential
- The application was regarded as an innovative tool, and health- and nutrition-conscious participants found it valuable. Participants proposed several ideas for further development, including integrating food-ordering features linked directly to restaurants, thereby streamlining the ordering process. In addition, participants recommended expanding the application's coverage to include more restaurants, allowing a wider audience to benefit. Furthermore, a user review feature was also suggested, enabling prospective diners to consider others' experiences before making their decisions.

## Conclusion

In this study, a DSS has been successfully developed for prioritizing restaurant food choices to make the best decision that suits individual consumers. It uses a GA that utilizes selection, crossover, mutation, and tournament selection across generations to help a diner find the best food combination provided in a restaurant. The uniqueness of this system is that it is based on data and information about food ingredients, nutrients, and calories and acts as a knowledge base to perform computational tasks to match them with the diner's nutrient and energy requirements with respect to their age, sex, weight, height, and NCD records. This approach follows the methodology of nutritional science and practice in recommending the best food for a diner. This system can be replicated or used in other restaurants by adding food and beverage data provided by the restaurants along with nutritional content information.

User feedback has shown that the system not only promotes nutritional awareness and supports healthy eating habits, especially among health-conscious individuals, but also offers ease of use and satisfaction across different age groups. Participants appreciated the convenience of automated nutrient calculations and value the application's informative features. Despite minor technical challenges, the overall user experience was positive, with many participants expressing enthusiasm and willingness to recommend the application to others. These findings reinforce the system's potential for contributing meaningfully to personalized nutrition practices.

Precision nutrition is a cutting-edge approach to diet and health that takes into account personal data, such as genetics, microbiome composition, and lifestyle factors, to create highly personalized nutrition plans. Future work on precision nutrition should focus on understanding and incorporating the diner's genetics, microbiome composition, and lifestyle factors into the system. Since the available knowledge on the existence of myriad microbiomes in a human body is still a research in progress, we concentrated specifically on the gut microbiome, which appears to influence many aspects of the overall health of humans, both within the digestive system and outside of it (39, 40). In addition, the microbiome is strongly related to foods and is individual-specific. Studies have investigated the associations between the microbiome and cholesterol levels, weight, blood glucose levels, and other clinical parameters. However, the associations between the F/B ratio of the microbiome and its function (41), such as antioxidants and probiotics, need to be further studied. The F/B ratio can be recorded and used to compute the suitability of foods consumed. Functional food ingredients that can potentially improve gut health include prebiotics, probiotics, synbiotics, and postbiotics. This will enable us to develop new specialized foods for patients with special needs in hospitals and those with diseases other than NCDs.

## Data Availability

The raw data supporting the conclusions of this article will be made available by the authors, without undue reservation.
